# Language Nativeness Modulates Physiological Responses to Moral vs. Immoral Concepts in Chinese–English Bilinguals: Evidence from Event-Related Potential and Psychophysiological Measures

**DOI:** 10.3390/brainsci13111543

**Published:** 2023-11-02

**Authors:** Fei Gao, Chenggang Wu, Hengyi Fu, Kunyu Xu, Zhen Yuan

**Affiliations:** 1 Institute of Modern Languages and Linguistics, Fudan University, Shanghai 200433, China; feigao@fudan.edu.cn; 2 Centre for Cognitive and Brain Sciences, University of Macau, Macau SAR 999078, China; 3 Key Laboratory of Multilingual Education with AI, School of Education, Shanghai International Studies University, Shanghai 200083, China; chenggangwu@outlook.com; 4 Institute of Linguistics, Shanghai International Studies University, Shanghai 200083, China; 5 Faculty of Health Sciences, University of Macau, Macau SAR 999078, China; yc27637@umac.mo

**Keywords:** moral concept, bilingual processing, emotion, N400, LPC

## Abstract

Morality has been an integral part of social cognition and our daily life, and different languages may exert distinct impacts on human moral judgment. However, it remains unclear how moral concept is encoded in the bilingual brain. This study, therefore, aimed to explore the emotional and cognitive involvement of bilingual morality judgement by using combined event-related potential (ERP) and psychophysiological (including skin, heart, and pulse) measures. In the experiment, thirty-one Chinese–English bilingual participants were asked to make moral judgments in Chinese and English, respectively. Our results revealed increased early frontal N400 and decreased LPC in L1 moral concept encoding as compared to L2, suggesting that L1 was more reliant on automatic processes and emotions yet less on elaboration. In contrast, L2 moral and immoral concepts elicited enhanced LPC, decreased N400, and greater automatic psychophysiological electrocardiograph responses, which might reflect more elaborate processing despite blunted emotional responses and increased anxiety. Additionally, both behavioral and P200 data revealed a reliable immorality bias across languages. Our results were discussed in light of the dual-process framework of moral judgments and the (dis)embodiment of bilingual processing, which may advance our understanding of the interplay between language and morality as well as between emotion and cognition.

## 1. Introduction

Morality denotes the fundamental principles that distinguish good from bad and right from wrong, which has been an integral part of social cognition and daily life in the form of moral beliefs, behaviors, and concepts, among others [1,2]. Recently, the relationship between morality and language has been extensively discussed (e.g., [3,4]), as language implicates a crucial tool and carrier of cognition and thinking, and meanwhile, different languages might exert distinct impacts on human thought [5,6,7]. This issue has aroused extensive attention from bilingual researchers, as it is intriguing whether bilingual participants would adopt differing morality standards, values, and beliefs when making moral judgments in their first (L1) and second languages (L2). In particular, the Moral Foreign-Language Effect [8,9] described the case in which bilinguals are more likely to make a utilitarian response (i.e., sacrifice one person’s life to save the other five) when the trolley dilemma is presented in their L2s as compared to in their L1s [10,11]. This effect has been interpreted as reduced emotion and/or increased cognitive load when using a foreign language; however, no consensus has been reached and it thus needs further validation [10,12,13,14]. More importantly, even though moral judgment among bilinguals has been extensively examined by using moral dilemmas, the cognitive basis of morality encoding at the concept level (i.e., abstract moral concepts like “fairness” and “dishonesty”) across L1s and L2s remains poorly understood. The present study, therefore, seeks to investigate whether and how L1 and L2 employ distinct cognitive and/or emotional resources to encode moral concepts in the bilingual population’s brain.

Recent meta-analyses reported a reliable foreign language effect on moral dilemma judgments and other decision-making processes like risk-taking and the “hot hand” effect [9,15,16,17], which was also arguably moderated by stimulus type, language proficiency, and L1–L2 similarity, among others. Across those studies, the dual-process framework has been applied to accommodate the differing strategies employed by L1 and L2 during decision-making processes [12,18]. Within this framework, automatic processing is, to a great extent, associated with emotional involvement and heuristic thinking, while controlled processing tends to rely on rational and deliberate analysis. In moral dilemma scenarios, specifically, the tendency of making utilitarian rather than deontological responses in L2 could be attributed to either reduced automatic/emotional processing or increased deliberation. However, the results were mixed with regard to the relative weights of emotion and cognition (e.g., [19,20,21]). The discrepancy might be due to the fact that those studies primarily based their findings on pen-and-paper-based or online questionnaires, which thus was limited in providing direct evidence regarding the underlying cognitive mechanism of moral decision making.

To address this limitation, a line of research made attempts to examine the extent of emotional and cognitive involvement in moral decision making and bilingual processing by utilizing neuroimaging and psychophysiological measures [13,22,23,24,25]. For instance, the event-related potential (ERP) technique draws on a high temporal resolution and measures the scalp electricity on a millisecond-by-millisecond basis while people are performing cognitive tasks [26,27], which can help decode the sub-processes and time courses of specific cognitive events. In particular, Liu et al. [28] examined the foreign language effect on altruistic decision making among Chinese–English bilinguals by using fused behavioral and ERP methods. Their behavioral results showed that participants were more likely to make altruistic decisions in the harm frame than the help frame when using L1 Chinese, which was, however, absent in their L2 English. In addition, the automatic N1 and N2 components were found to be associated with a framing effect (harm vs. help frames) in L1, yet not in L2, which was thus interpreted as a reduced emotion effect. Similar results were also obtained by Liu, Schwieter, Wang and Liu [24] using lexical–semantic judgment and gambling tasks, in which the rationality of making decisions in L1 Chinese after negative words was reduced yet enhanced in L2 English, as manifested by the P3 effect.

Meanwhile, multiple psychophysiological measures have been increasingly used to examine the (dis)embodied processing of language and emotion, including facial motor resonance, skin conductance, and heart rate [13,29,30,31,32,33]. Specifically, increased facial motor resonance and skin conductance values were detected for emotional words in L1 [30,31], which was supportive of an embodied view of language [34]. As conceptual knowledge (e.g., emotion, morality) is grounded in internal sensorimotor systems, thinking and making decisions in L2 may activate less sensorimotor knowledge and emotional engagement as compared to that in L1. As such, we attempted to explore the emotional and cognitive involvement of bilingual morality judgement at the concept level by using combined ERP and psychophysiological measures.

The moral concept constitutes important conceptual knowledge in our social life [35]. Human beings use moral concepts to categorize behaviors into good vs. bad, right vs. wrong, and virtue vs. vice. Haidt and Algoe [1] suggested that the dissociation and amplification between good and evil implicates an integral part of social cognition, which is especially pronounced in the early development of the sense of moral bipolar judgement. This dissociation might lead to an “immorality bias”, such that immoral concepts (e.g., dishonest, evil) tended to be more salient than moral ones (e.g., caring, charity). Consequently, the recognition of immoral traits needs increased attention and thus takes longer reaction times [36,37], which is considered an evolutionarily adaptive strategy [2].

Existing studies on moral concept encoding in humans concentrated on the metaphorical representations of moral traits, including space [36,37], cleanliness [38,39], brightness [37], and physical forms [35]. Compared to immoral concepts, moral concepts were recognized faster when presented at a higher position on the computer screen, in non-distorted fonts, and with better self/environmental cleanliness. These studies thus shed light on the notion that moral concepts might be primarily comprehended through grounded concepts and their embodiment in the physical world. However, far too little attention has been paid to the cognitive mechanisms of moral concept encoding in the human brain. This issue becomes even more intriguing when it comes to bilingualism. As L2 learning and bilingualism have become an international phenomenon, people inevitably encode moral concepts and make moral decisions when using an L2. This indicates a need to understand the emotional and cognitive involvements underlying moral concept encoding in L1 and L2 within a bilingual brain.

Therefore, the current study aims to investigate whether bilinguals will employ similar or distinct cognitive and/or emotional resources when encoding moral concepts in their L1s and L2s. To examine the (dis)embodiment and phycological distance between L1 vs. L2 and moral vs. immoral traits, we simultaneously recorded behavioral data and electrophysiological signals (including scalp, skin, heart, and pulse) when Chinese–English bilingual participants were making moral judgments. We hypothesized that immoral concepts would be linked to increased salience and attention (e.g., longer reaction times and greater physiological responses) than moral concepts across languages. Meanwhile, L1 Chinese might elicit enhanced emotion (early ERP components like P200 and P300, e.g., [22,23]) and less elaboration (e.g., Late Positive Component, LPC, [40,41,42]) as compared to L2 English, in light of the dual-process framework. Our results would adduce the neuro-scientific evidence of the moral foreign language effect and advance our understanding of the interplay between morality and language.

## 2. Methods

### 2.1. Participants

Thirty-one college students (15 females, mean age: 23.5 years; SD = 3.2) from the University of Macau participated in the experiment for monetary compensation. All participants were right-handed bilinguals with Chinese as their L1s and English as their L2s. They had normal or corrected-to-normal vision and no neurological or psychological illness. All participants were born in mainland China and started to learn L2 English at a mean age of 7.42 years old (SD = 2.63) through classroom instructions. Their English proficiency was measured by using the Lexical Test for Advanced Learners (LexTALE), which is a brief and valid test for assessing L2 English vocabulary and general proficiency [43]. All participants obtained an average LexTALE score of 63.37 out of 100 (SD = 7.44), indicating intermediate English proficiency in general. The study was granted ethical approval by the Institutional Review Board of the University of Macau. An informed consent form was obtained from each participant prior to the experiment.

### 2.2. Materials

Forty-six English words with a moral meaning (e.g., kindness, truth, donate) and forty-six words with an immoral meaning (e.g., murder, killer) were selected from a dataset of 13,915 English emotional lemmas [44], with these words’ valence and arousal values retrieved. Likewise, 46 Chinese words with a moral meaning (e.g., 仁爱, benevolence) and 46 words with an immoral meaning (e.g., 小偷, thief), as well as their valence and arousal ratings were retrieved from a Chinese dataset of 1100 words [45]. The selection procedure and outcomes were completed and approved by three experts in linguistics. To verify the grouping of moral/immoral concepts, we asked 20 college students to complete a morality-rating survey on the 92 English words and 92 Chinese words, where 1 indicated “definitely immoral”, while 9 indicated “completely moral”. The results showed that moral words manifested significantly higher moral meanings (English: 7.14 ± 0.59; Chinese 7.28 ± 0.57) than immoral words (English: 2.11 ± 0.59; Chinese: 1.88 ± 0.50) (*ps* < 0.01). Another 16 college students were invited to rate their familiarity with these words using a 9-point scale, which showed high familiarity (English: >7.9; Chinese: 9). Valence values were significantly higher in moral concepts than in immoral concepts (*ps* < 0.05). Arousal values, frequency, word length (including stroke number), and familiarity were matched across moral vs. immoral concepts (*ps* > 0.05) and across English vs. Chinese concepts (*ps* > 0.05). Examples and relevant statistics of the four conditions are demonstrated in Table 1. In particular, English word frequency information (log10) was retrieved from the SubtlexUS dataset [46], and Chinese information was obtained from the Subtlex-CH [47]. All stimuli and data are available online (https://osf.io/sxrkf/, created on 8 October 2023).

### 2.3. Procedure

All materials were divided into practice sessions: 2 Chinese blocks, and 2 English blocks. Each block contained 23 moral concepts and 23 immoral concepts. There were 10 Chinese trials and 10 English trials for practice, half moral and the other half immoral, right before the Chinese and English tasks, respectively. Only when the participants obtained a perfect score in the practice session could they proceed to the experimental blocks. Then, they were invited to take morality decision tasks in Chinese (2 blocks) and English (2 blocks), respectively, while electroencephalogram (EEG), electrocardiograph (ECG), electrodermal activity (EDA), and photoplethysmogram (PPG) signals were recorded at the same time. The order of Chinese and English tasks was randomized among all participants, while the two blocks of the same language were performed consecutively to minimize the potential language switch costs. All materials were coded by using E-prime 3.0 and presented in white against a black background. The Chinese words were in SimSun font, and the English font was Times New Roman at a size of 40 points. In each trial, participants would first encounter a fixation lasting for 800 ms, followed by the target word of Chinese/English concepts. They needed to decide whether this concept was moral or not by pressing the corresponding buttons on the keyboard within 3 s. Otherwise, this trial would be marked as incorrect by the software. After the participants’ response or after 3 s, there would be a blank whose duration jittered from 800 to 1000 ms.

### 2.4. Psychophysiological Recordings and Analyses

EEG data, along with EDA, PPG, and ECG, were recorded when participants were conducting the morality judgment tasks. EEG signals were continuously acquired at a rate of 2048 Hz from 32 electrodes of the Biosemi EEG system in light of a 10–20 convention. Impedances were kept below 5 kΩ for each electrode. The offline EEG data were analyzed by using EEGLAB v2021.0. Each dataset was down-sampled to 500 Hz, re-referenced to the mean voltage of the whole scalp, and then filtered with a band-pass of 0.01–30 Hz with a Butterworth filter. The filtered data were then segmented into epochs ranging from −200 to 800 ms after the onset of moral and immoral concepts. Subsequently, ocular artifacts, including blinks and horizontal eye movement, were removed by using an independent component analysis (ICA). On average, 1.58 ± 0.72 components were removed from each participant. In addition, bad channels were interpolated by using a spheric method, while data with voltages over ±100 μV would be marked as artifacts and removed from further analysis.

In light of visual inspection and previously related studies [23,25,48], we focused on three ERP components [P200 (150~200 ms), N400 (250~400 ms), and Late Positive Component (LPC, 400~700 ms)] to examine the language and morality effects during moral concept encoding. In particular, P200 effect was examined in the frontal sites (F3, Fz, and F4) with a three-way ANOVA of language (Chinese vs. English), morality (moral vs. immoral), and laterality (left: F3; midline: Fz; right: F4). Meanwhile, N400 and LPC effects were quantified by using four-way ANOVA with language, morality, region (frontal: F3, Fz, F4; central: C3, Cz, C4; parietal: P3, Pz, P4), and laterality (left: F3, C3, P3; midline: Fz, Cz, Pz; right: F4, C4, P4) as factors.

Additionally, participants’ heart rate was measured by using three electrodes connected to the right wrist (negative), the left ankle (positive), and the right ankle (ground) with the electrocardiograph (ECG) module of the MP160 BIOPAC system. Skin conductance was recorded by the electrodermal activity (EDA) electrodes attached to the index and middle fingers of the participants’ left hand. Additionally, participants’ pulse changes were measured using the photoplethysmogram (PPG) module, whose sensor was connected to the ring finger of the left hand. ECG, EDA, and PPG data were sampled at 250 Hz and analyzed with a reference of a 5 min resting before the formal test.

## 3. Results

### 3.1. Behavioral Results

All participants performed an average accuracy (ACC) of 96% among all formal morality judgement tasks, which verified their familiarity and engagement with those materials. Given the low signal-to-noise ratio and the superimposing issue of ERP as well as existing studies [49,50], we adopted a linear mixed-effect model for behavioral data only, while ERP data were analyzed by using repeated-measures ANOVA.

Both reaction time (RT) and ACC data were analyzed using a linear mixed-effect model using the lme4 package and ANOVA function in RStudio 4.3.0. Language (Chinese vs. English) and morality (moral vs. immoral) served as fixed factors, while participants and items were treated as random factors. A maximal random-effects structure was used by including random slopes of the participants and items [51]. The formulas of modelling and comparisons are available on OSF (https://osf.io/sxrkf/, created on 8 October 2023). The RT results revealed a significant main effect of language (*F* = 133.728, *p* < 0.001), such that Chinese concepts were associated with faster responses than English concepts. The main effect of morality was also significant (*F* = 11.625, *p* < 0.001). Moral concepts were responded to faster than immoral concepts. Meanwhile, there was a significant interaction between language and morality (*F* = 6.117, *p* < 0.05). The following comparisons showed the same results as the main effect.

**Figure 1:**
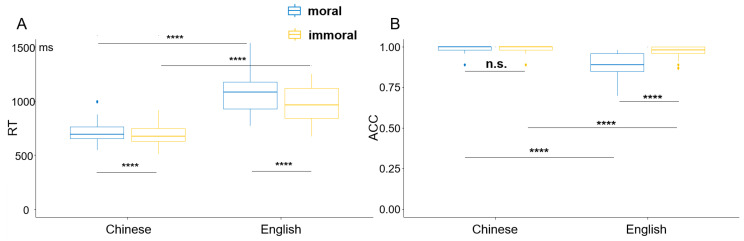
Behavioral results of the morality judgment task among all participants. (**A**) RT results across the four conditions. (**B**) ACC results across the four conditions. **** denotes *p* < 0.0001, “n.s” means “not significant”.

The ACC results revealed significant main effects of language (*F* = 30.864, *p* < 0.01) and morality (*F* = 14.326, *p* < 0.01). Chinese tasks obtained significantly higher accuracy than English ones, and moral concepts reached higher accuracy than immoral concepts. There was a significant interaction between language and morality (*F* = 2.623, *p* < 0.01), such that the moral vs. immoral difference was significant in English (*p* < 0.001) rather in Chinese (*p* = 0.415). RT and ACC results are visualized in Figure 1.

### 3.2. EEG Results

#### 3.2.1. P200 Results

**Figure 2:**
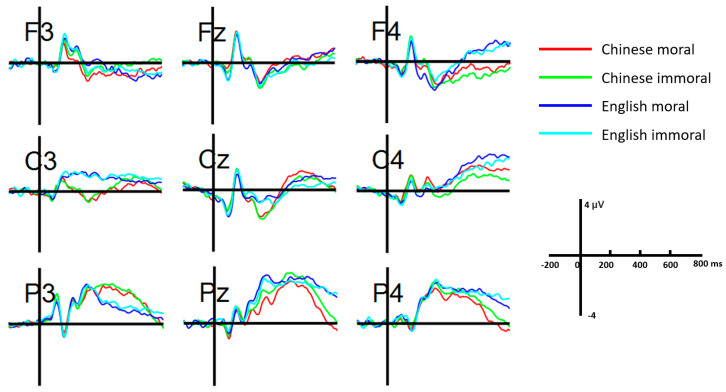
Averaged ERP waveforms at representative sites across the four conditions.

**Figure 3:**
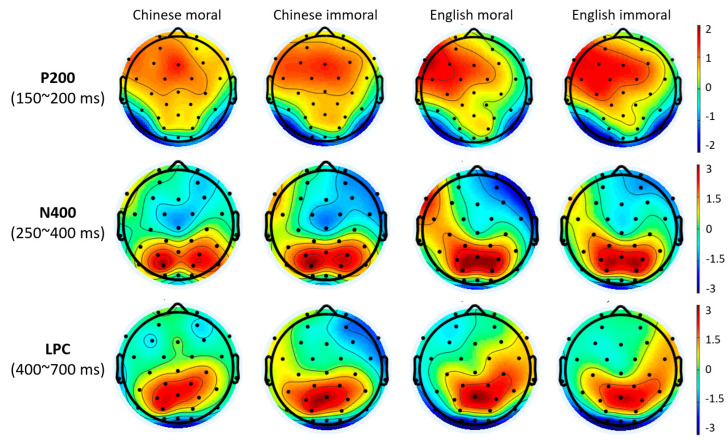
Scalp distribution of voltage in the time windows of P200 (150~200 ms), N400 (250~400 ms), and LPC (400~700 ms).

As can be seen from Figure 2 and Figure 3, P200 was pronounced at the frontal sites F3, Fz, and F4. The three-way repeated-measures ANOVA indicated a significant morality effect (*F*(1, 30) = 5.869, *p* = 0.022, partial *η*^2^ = 0.164), such that immoral (1.3 ± 0.36 μV) concepts elicited greater positivity than moral ones (1.03 ± 0.39 μV). No other significant main effect or interaction was identified (*ps* > 0.05).

#### 3.2.2. N400 Results

**Figure 4:**
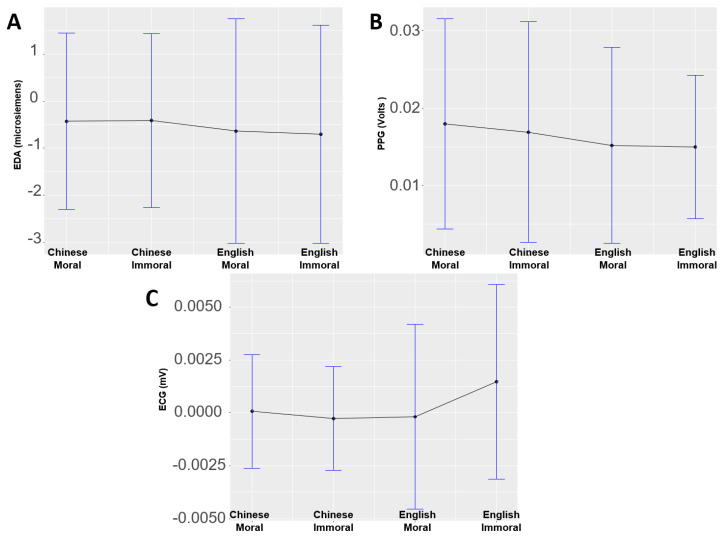
Psychophysiological results across the four conditions. (**A**) EDA results. (**B**) PPG results. (**C**) ECG results.

N400 was mainly distributed at the frontal and central cortices (Figure 3 and Figure 4). There was a significant main effect for language (*F*(1, 30) = 7.807, *p* = 0.009, partial *η*^2^ = 0.206). Chinese concepts (0.19 ± 0.38 μV) were associated with enhanced N400 as compared to English concepts (0.69 ± 0.36 μV). There was also a significant effect for brain region (*F*(2, 60) = 18.857, *p* < 0.001, partial *η*^2^ = 0.386), such that the frontal and central sites elicited greater negativities than the parietal sites (*ps* < 0.001), while there was no significant difference between the frontal and central sites (*p* = 0.179). In addition, we identified a significant three-way interaction between language, morality, and laterality (*F*(2, 60) = 3.484, *p* = 0.037, partial *η*^2^ = 0.104), such that Chinese concepts elicited greater N400 than English ones for both moral and immoral conditions in the left hemisphere. There was also a significant three-way interaction between language, region, and laterality (*F*(4, 120) = 4.406, *p* = 0.002, partial *η*^2^ = 0.128), where Chinese concepts generated enhanced N400 than English ones at the central sites of the left hemisphere as well as the midline sites of the posterior region. Meanwhile, there was a significant four-way interaction (*F*(4, 120) = 6.422, *p* < 0.001, partial *η*^2^ = 0.176). The following comparisons showed that Chinese moral concepts elicited greater negativities than Chinese immoral ones at the midline site of the posterior cortex, while English moral concepts generated enhanced negativities than English immoral ones at the frontal sites of the right hemisphere. No other significant main effect or interaction involving language or morality was identified (*ps* > 0.05).

#### 3.2.3. LPC Results

There was a significant region effect on LPC (*F*(2, 60) = 6.771, *p* = 0.002, partial *η*^2^ = 0.184), such that positivities at the posterior sites were significantly greater than those of the frontal and central sites (*ps* < 0.05). The results also revealed a significant interaction between language and laterality (*F*(2, 60) = 4.673, *p* = 0.013, partial *η*^2^ = 0.135). Simple effects analysis showed that English concepts generated greater LPC than Chinese ones in the right hemisphere. Meanwhile, there was a significant three-way interaction between language, region, and laterality (*F*(4, 120) = 2.667, *p* = 0.036, partial *η*^2^ = 0.082), such that English concepts were associated with greater positivities than Chinese ones at the frontal sites of the right hemisphere. There were no other significant interaction effects related to language or morality (*ps* > 0.05).

### 3.3. EDA, PPG, and ECG Results

Two-way ANOVA with language and morality as factors were conducted on EDA, PPG, and ECG data, respectively. The results (Figure 4) indicated a significant language effect on ECG (*F*(1, 30) = 4.67, *p* < 0.05, partial *η*^2^ = 0.135), while no other main effect or interaction was significant (*ps* > 0.05).In particular, English concepts elicited significantly higher ECG values than Chinese ones. However, there were no significant main effects or interactions on EDA and PPG data (*ps* > 0.05).

## 4. Discussion

By using behavioral and electrophysiological (including ERPs, skin, heart, and pulse), our study explored the cognitive and neural correlates of bilingual moral concept encoding. Our results added novel neural and psychophysiological evidence that verified the (dis)embodiment and psychological distance between L1 and L2 moral vs. immoral concepts.

First, both RT and ACC data of our study showed a pronounced “immorality bias” effect, in line with studies on the vertical representation of English moral concepts [36,37]. While existing studies suggested that cultural and linguistic backgrounds might modulate people’s moral decision making [4,17], our behavioral results indicated that immorality bias was persistent across languages. For both L1 Chinese and L2 English within the bilingual brain, immoral concepts were recognized more slowly and more accurately than moral concepts. The increased difficulty with immorality could be attributed to an enhanced attention to its salience, which was a result of adaptive behavior during the evolutionary process. People would choose to avoid those who betrayed them and with immoral traits [2,37]. This would significantly affect people’s moral decisions in social life throughout their lifespan.

Meanwhile, Chinese concepts (for both moral and immoral) were associated with faster responses and higher accuracy rates as compared to English concepts. This language effect might relate to the participants’ language experience and proficiency. The participants we recruited were late bilinguals (age of acquisition: around 7.42 years old) with an average intermediate proficiency of L2 English. As such, their performance in L2 would be unlikely to reach a native-like status. Nevertheless, we speculated that the confounding effect from L2 proficiency was minimal in our study, as the ACC in L2 English was pretty high (around 92.5%) and thus indicated participants’ good engagement in the task. The interaction between language and morality in ACC data showed that the moral vs. immoral difference was absent in L1 Chinese, which could be attributed to a ceiling effect in terms of native language. Further, ERP and psychophysiological measures could provide direct evidence on the sub-processes and temporal signatures of language effects and immorality bias.

Our ERP results first revealed a frontal P200 effect between moral and immoral concepts for both L1 Chinese and L2 English. The enhanced P200 elicited from immoral concepts verified the immorality bias manifested by behavioral data from a neurobiological perspective. In particular, P200 has been recognized as an early index of attention orientation to the emotional content of stimuli [23,41]. Related studies showed that emotional words (positive/negative) elicited greater P200 than neutral words [52,53]. Our P200 findings thus extended the emotional content to morality and further confirmed the increased salience and attention associated with immoral concepts. Interestingly, the language effect detected from behavioral results was not identified from the frontal P200. We took similar P200 patterns between L1 and L2 as evidence of comparable competence across languages. Bilinguals might not encounter additional difficulties for L2 morality judgment, so they do not need to pay extra attention to the stimuli.

Following P200, we identified an N400 component mainly distributed at the frontal sites of the scalp. Yet, the negativities we obtained showed slight inconsistency with the classic semantic N400 effect at the centro–parietal cortex [54,55]. Those typical N400 were primarily associated with semantic violations and thus could index conscious lexico–semantic processing. In contrast, the semantic involvement of this early N400 in our study might be minimal at best since L1 Chinese elicited enhanced negativities than L2 English. Even though the L2 task obtained high accuracy, it was not likely that L2 could outperform L1 regarding semantic strategies. We thus interpreted this early N400 at the frontal sites as an index of emotional salience. Interestingly, Jonczyk, Boutonnet, Musial, Hoemann and Thierry [25] reported a similar N400 effect when Polish–English bilinguals were reading L1 and L2 sentences embedded with affectively (in)congruent words. Greater N400 was found with L1 Polish sentences, while reduced N400 was detected for negative statements in L2 English. As such, the early N400 effect suggested increased emotional experience in L1 and reduced emotional embodiment of L2 in moral judgments, which was supportive of the intuitive and automatic route of the dual-process framework of moral judgments [12,18]. Meanwhile, the functional role of our early N400 resembled the early posterior negative (EPN) of previous ERP studies [56,57] on emotional word processing and depicted an early automatic and implicit processing of emotional information. Especially for L2 English, semantic access to (im)moral concepts might be suppressed at the early time window of semantic integration. This would call for an elaborate processing at later stages.

In particular, both moral and immoral concepts of L2 English elicited increased negativities at 400~700 ms, particularly at the posterior sites, as compared to L1 Chinese. This line of finding was consistent with the well-documented LPC or LPP (late positive potential), which links to the controlled and explicit processing [40,41,48,58]. For instance, Naranowicz, Jankowiak, Kakuba, Bromberek-Dyzman and Thierry [42] detected increased LPC in L2 English sentence reading after viewing negative-valenced affective films as compared to L1 Polish among unbalanced Polish–English bilinguals. They interpreted this language effect as more demanding semantic integration and re-analysis with L2. Likewise, the enhanced LPC we identified from L2 moral and immoral concepts relative to L1 would link to increasing cognitive load from using a foreign language. This finding corroborated the controlled and elaborated route of the dual-process account moral judgments. Even though L2 moral and immoral concepts induced less emotional engagement, bilingual individuals would pay extra effort and use elaborate strategies to compensate for the emotional shortage to complete the morality evaluation. More importantly, even though the process dissociation account suggested the minimal role of elaboration in moral judgments [20,21], our findings suggested that emotion and elaboration were not mutually exclusive in L2 morality encoding.

In addition, we found greater automatic psychophysiological activities with electrocardiographs in L2 moral judgments than in L1 Chinese, which is inconsistent with previous bilingual studies using skin conductance measures [30]. We tended to interpret the differential automatic activities across languages from our study to the differing anxiety levels when making decisions in different scenarios [32]. It is likely that the increased anxiety in L2 would link to greater psychological distance, which further corresponds to the elaborate processing at the later stage of morality judgment. This notion, however, needs verification in future studies.

Taken together, the current study shed light on a prominent embodiment in L1 and a less grounded cognition in L2 during bilingual morality encoding. Every human being acquired his or her native language in natural settings enriched with affective stimuli and embodied experiences, while L2s were learned more explicitly with formal instruction and later in life. This distinction would lead to differing minds and thinking in terms of L1 and L2, including the decision of good from bad and right from wrong. As a consequence, moral judgment in L1 would be more reliant on automatic processes and emotions and less on elaboration and rationality, as manifested by increased early N400 and decreased LPC. In contrast, L2 moral and immoral concepts elicited enhanced LPC, decreased N400, and greater automatic psychophysiological responses of electrocardiograph, which might denote more elaborate processing yet blunted emotional responses and increased anxiety. Meanwhile, both L1 and L2 revealed a reliable immorality bias, as manifested by both behavioral and P200 data, which constituted an important adaptative behavior during evolutionary processes.

## 5. Conclusions

This study is among the first to investigate the behavioral and neural correlates of bilingual morality encoding at the concept level. We adduced neuro-scientific evidence to the (dis)embodied processing of L1 and L2 morality within the bilingual brain and the immorality bias. Our findings will advance our understanding of the interplay between language and morality as well as between emotion and cognition. Future research is needed to extend this exploratory study and put moral concepts in sentential contexts and various metaphorical representations.

## Figures and Tables

**Table 1:** The example, emotional, and lexical properties among the four conditions. Standard deviations are displayed in parentheses.

Language	Type	Example	Valence	Arousal	Frequency	Word Length	Stroke Number	Morality	Familiarity
English	Moral	honesty	6.61 (0.87)	4.12 (0.91)	2.74 (0.61)	7.59 (1.95)	/	7.14 (0.59)	8.39 (0.38)
	Immoral	murder	2.6 (0.60)	5.46 (0.77)	2.80 (0.64)	6.50 (1.99)	/	2.11 (0.59)	7.51 (0.84)
Chinese	Moral	正义, justice	5.46 (1.99)	5.99 (0.83)	2.44 (0.76)	2	16.41 (5.26)	7.28 (0.57)	9
	Immoral	拐骗, abduct	3.91 (1.99)	5.62 (1.07)	2.22 (0.82)	2	17 (4.26)	1.88 (0.50)	9

## Data Availability

Data are available upon request.
