# Correction: Gao et al. Language Nativeness Modulates Physiological Responses to Moral vs. Immoral Concepts in Chinese–English Bilinguals: Evidence from Event-Related Potential and Psychophysiological Measures. *Brain Sci.* 2023, *13*, 1543

**DOI:** 10.3390/brainsci16030262

**Published:** 2026-02-26

**Authors:** Fei Gao, Chenggang Wu, Hengyi Fu, Kunyu Xu, Zhen Yuan

**Affiliations:** 1 Institute of Modern Languages and Linguistics, Fudan University, Shanghai 200433, China; feigao@fudan.edu.cn; 2 Centre for Cognitive and Brain Sciences, University of Macau, Macau SAR 999078, China; 3 Key Laboratory of Multilingual Education with AI, School of Education, Shanghai International Studies University, Shanghai 200083, China; 4 Institute of Linguistics, Shanghai International Studies University, Shanghai 200083, China; 5 Faculty of Health Sciences, University of Macau, Macau SAR 999078, China

## Text Correction

In the Discussion section of the original publication [1] (Section 4, second paragraph) the sentence “immoral concepts were recognized more slowly and more accurately than moral concepts” contains a typo.

It should read “immoral concepts were recognized more slowly and less accurately than moral concepts.”

## Figure Correction

In Figure 1A,B, the color coding for moral and immoral was inadvertently reversed. See the updated Figure 1 below. 

The scientific conclusions are unaffected.

**Figure 1:**
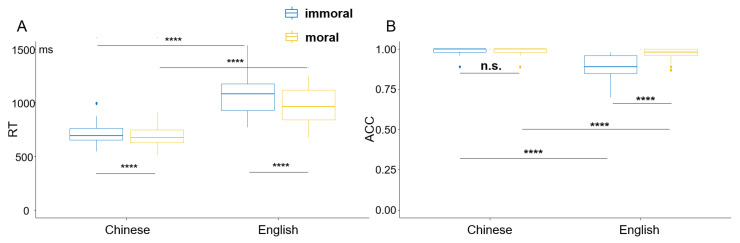
Behavioral results of the morality judgment task among all participants. (**A**) RT results across the four conditions. (**B**) ACC results across the four conditions. **** denotes *p* < 0.0001, “n.s.” means “not significant”.
